# Are wearable devices effective for preventing and detecting falls: an umbrella review (a review of systematic reviews)

**DOI:** 10.1186/s12889-021-12169-7

**Published:** 2021-11-14

**Authors:** Daniel Joseph Warrington, Elizabeth Jane Shortis, Paula Jane Whittaker

**Affiliations:** Division of Population Health, Health Services Research & Primary Care, Faculty of Biology, Medicine and Health, University of Manchester, Room 2.545, Stopford Building, Oxford Road, Manchester, M13 9PT UK

**Keywords:** Wearable electronic devices, Accidental falls, Aged, Falls prevention, Falls management

## Abstract

**Background:**

Falls are a common and serious health issue facing the global population, causing an estimated 646,000 deaths per year globally. Wearable devices typically combine accelerometers, gyroscopes and even barometers; using the data collected and inputting this into an algorithm that decides whether a fall has occurred. The purpose of this umbrella review was to provide a comprehensive overview of the systematic reviews on the effectiveness of wearable electronic devices for falls detection in adults.

**Methods:**

MEDLINE, Embase, Cochrane Database of Systematic Reviews (CDSR), and CINAHL, were searched from their inceptions until April 2019 for systematic reviews that assessed the accuracy of wearable technology in the detection of falls.

**Results:**

Seven systematic reviews were included in this review. Due to heterogeneity between the included systematic reviews in their methods and their reporting of results, a meta-analysis could not be performed. Most devices tested used accelerometers, often in combination with gyroscopes. Three systematic reviews reported an average sensitivity of 93.1% or greater and an average specificity of 86.4% or greater for the detection of falls. Placing sensors on the trunk, foot or leg appears to provide the highest accuracy for falls detection, with multiple sensors increasing the accuracy, specificity, and sensitivity of these devices.

**Conclusions:**

This review demonstrated that wearable device technology offers a low-cost and accurate way to effectively detect falls and summon for help. There are significant differences in the effectiveness of these devices depending on the type of device and its placement. Further high-quality research is needed to confirm the accuracy of these devices in frail older people in real-world settings.

## Background

### Description of the condition

Falls are a common and serious health issue facing the global ageing population [1]. Globally, falls are the second leading cause of unintentional death injury after road traffic accidents, causing an estimated 646,000 deaths each year [2]. Frequency of falls increases with age and increased fragility, with studies showing that up to 28–35% of adults over the age of 64 falls every year [3]. This equates to a serious human cost including loss of independence, pain, and mortality.

In addition to the physical impact of falling, falls can cause post-fall anxiety syndrome (fear of falling) [4]. This can lead to a lack of confidence in older people in their ability to walk safely, resulting in self-imposed activity restrictions leading to further decline in both their physical and mental health [5].

A 2013 systematic review and meta-analysis (Deandrea et al. 2013) showed that there are several strong predictors for falls risk: a history of falls, use of walking aids and disability [6]. Accurate identification of those at risk of falls is important so that interventions that detect falls can be targeted appropriately.

### Description of the intervention

A 2017 Cochrane review identified exercise programmes, and multifactorial interventions integrating assessment with individualised intervention and home safety interventions (i.e. anti-slip shoes) as the most effective interventions for preventing falls in older people [7]. The National Institute for Clinical Excellence (NICE) in England’s guidance on falls prevention does not mention any technological interventions [8]. Despite current interventions to prevent falls, this public health challenge demands innovate solutions due to its debilitating effect on the quality of life of older adults. Age UK advocates the use of telecare for falls detection [9].

Wearable technology for falls detection is an emerging technology. This wearable technology typically includes an accelerometer and an algorithm with some more complicated sensors including barometric sensors [10]. These systems are commonly used due to their low cost and relatively high sensitivity; however, it is important to consider which type of technology to use and their location on the body [11]. These sensors range from sensors in shoes to sensors that you can wear on your wrist, forearm, waist, pelvis, neck, sternum, chest, thigh, cruris, shank, knee and ankle [11]. These sensors typically use the data collected by the accelerometer or barometer and input them into an algorithm that decides whether a fall has occurred [12]. Once the device has decided that a fall is likely, current devices are usually designed so that this triggers an alert (phone call, text message, email) to a nominated person, caregiver or emergency service so that they can receive medical attention [13]. Apart from this alert-based falls detection approach, these types of sensors have also been used as part of falls risk assessments to help assess how at risk an individual is of falling so that effective, targeted interventions can be prescribed to that individual [14].

### Why it is important to do this review

Falls detection is a widely researched topic with several systematic reviews published in the last 5 years. Recent systematic reviews on the use of wearable technology for falls have examined the most effective type of these sensors for falls detection, their use in older adults, their use in Parkinson’s disease and their use in detecting near falls [10, 11, 13–17].

An umbrella review is required to summarise the evidence of the ability of wearable electronic devices to detect falls accurately and to guide further research in this field.

### Aims

The aim of this umbrella review was to complete an umbrella review of the literature on the effectiveness of wearable electronic devices for falls detection in adults. All outcomes in included systematic reviews will be considered including falls detection, falls prevention, assessing the risk of falling, reduction in hospital admission and reduction in fractures due to falls.

## Methods

### Registry of umbrella review protocol

This review followed the Preferred Reporting Items for Systematic Reviews and Meta-Analyses (PRISMA) guidelines [18]. The review protocol was established prior to the conduct of the review and was registered at the International Prospective Register of Systematic Reviews (PROSPERO) (registration number CRD42019133954 – available from: http://www.crd.york.ac.uk/PROSPERO/display_record.php?ID=CRD42019133954).

### Literature search

Four electronic databases, MEDLINE, Embase, Cochrane Database of Systematic Reviews (CDSR), CINAHL, were searched from their inceptions until April 2019. Articles were searched using Boolean combinations of the following keywords or equivalent Medical Subject Heading (MeSH) terms: accidental falls AND (wearable electronic devices OR wearable technology OR wearable device OR wearable sensor OR smartwatch). The searches were limited to include systematic reviews only according to the Scottish Intercollegiate Guidelines Network (SIGN) grading system for systematic reviews [19]. No language or other restrictions were applied to the initial search. The main search strategy can be found in Appendix 1**.** A full electronic search strategy for each database is available on request.

A grey literature search was conducted by searching OpenGrey and Google search engines. The reference lists of the included studies were searched, and a forward citation search was conducted of all included studies to identify any further relevant reviews. The following topic expert groups were contacted to request requests from any unpublished or yet to be published reviews: Age UK, National Falls Prevention Coordination Group (NFPCG) and Public Health England, National Falls Prevention Coordination Group.

### Inclusion criteria

Papers were considered suitable for this review if they met all the following criteria. Reviews must be original systematic reviews or meta-analyses with no date of publication limits. Articles must be published in English with the full-text article available. Articles must use adults (> = 18 years of age) with or without chronic disease (including Parkinson’s disease and stroke). Articles may include any intervention that is focussed on wearable electronic devices. Articles that measured reduction in falls (e.g., reduction in hospital admission, reduction in fractures, improved quality of life) or articles that measured the effectiveness of wearable technology in fall prevention or falls detection should be included.

### Paper selection and data extraction

Following the search strategy detailed above, titles and abstracts of the studies were screened independently according to the inclusion criteria by the 1st (DJW) reviewer and 2nd reviewer (EJS). The full texts of studies that were included based on titles and abstracts were retrieved and independently assessed for eligibility by the 2 reviewers. Any discrepancies between the 1st and 2nd reviewer were resolved by discussion with the 3rd (PJW) reviewer.

The following data were extracted independently by the 1st and 2nd reviewers, and checked for accuracy by the 3rd reviewer: number and year of publication of included studies, databases searched, review objectives, population characteristics, sample size, types of devices, main results, and outcome measures (see Table 1).

**Table 1:** Methodology of included systematic reviews and meta-analyses

Author	Number of and year of publication of included studies	Databases Searched	Study Objective	Population	Sample Size	Type of Device	Main Results
**Pang et al 2019** [17]	*N* = 9 (2010–2015)	CINAHL Embase MEDLINE Compendex	To summarise and critically examine evidence regarding the detection of near falls (slips, trips, stumbles, missteps, incorrect weight transfer, or temporary loss of balance) using wearable devices.	Adults (aged > = 18 years of age)	Average per study = 21 participants Total = 192 participants	*N* = 3 (accelerometer) *N* = 4 (accelerometer and gyroscope) *N* = 1 (accelerometer and an Android mobile phone) *N* = 1 (multiple sensors)	*N* = 5 (Accuracy/sensitivity and specificity of 97% or greater) *N* = 3 (Accuracy was improved by increasing the number of wearable devices) *N* = 2 (Chest and right thigh most accurate location for single device placement)
**Nguyen et al 2018** [13]	*N* = 24 (2015–2017)	Springerlink Elsevier IEE Xplore Digital Library Multidisciplinary Digital Publishing Institute (MDPI)	To systematically evaluate the use of Internet of Things (IoT) technology, especially in terms of sensing techniques and data processing techniques in performing falls management for supporting older adults to live independently and safely.	Adults (aged > = 18 years of age)	Average per study = 7 participants Total = 170 participants	*N* = 5 (accelerometer) *N* = 2 (accelerometer and gyroscope) *N* = 3 (smartphone) *N* = 6 (camera or laser) *N* = 2 (“wearable sensor”) *N* = 3 (multiple devices) *N* = 2 (wireless networks)	Wearable devices are effective for falls detection - achieving high specificity, sensitivity, and accuracy. Heterogenous methodology in the included studies make quantitative interpretation difficult.
**Montesinos et al 2018** [10]	*N* = 13 (2008–2014)	PubMed Embase IEEE Xplore Cochrane Central Registry of Controlled Trials (CENTRAL) ClinicalTrials.gov World Health Organisation International Clinical Trials Registry Platform	To synthetize the empirical evidence regarding inertial sensor-based falls risk assessment and prediction to identify optimal combination of sensor placement, task and features aiming to support evidence-based design of new studies and real-life applications.	At least 10 participants with an average age of 60 years old or over with no severe cognitive or motor impairment. Studies in which participants were labelled as fallers and non-fallers.	Average per study = 93 Total = 1211 participants	*N* = 9 (accelerometer) *N* = 3 (accelerometer and gyroscope) *N* = 1 (gyroscope)	The statistical analysis of features reported in the 13 shortlisted studies revealed significant, very strong, positive associations in 3 different triads of feature category, task, and sensor placement: • Angular velocity – Walking – Shins • Linear acceleration – Quiet standing – Lower back • Linear acceleration – Stand to sit/Sit to stand – Lower back
**Chaudhuri et al 2014** [16]	*N* = 57 (2007–2013)	PubMed CINAHL Embase PsycINFO	To systematically assess the current state of design and implementation of fall detection devices. This review also examines the extent to which these devices have been tested in the real world as well as the acceptability of these devices to older adults.	Adults (aged > = 18 years of age)	Information not available	*N* = 57 (wearable systems)	Most common types of devices: • Systems with device on trunk. Median sensitivity = 97.5% (range 81–100). Median specificity = 96.9% (range 77–100) • Systems involving multiple sensors. Median sensitivity = 93.4% (range 92.5–94.2) and a median specificity of 99.8% (range 99.3–100). • Systems involving devices around arms, hands, ears, or feet had a lower median sensitivity and specificity [81.5% (range 70.4–100) and 83% (range 80–95.7) respectively].
**Silva de Lima et al 2017** [15]	N = 4 (2005–2015)	PubMed Web of Science databases	To provide an overview of the use of wearable systems to assess freezing of gait (FOG) and falls in Parkinson’s disease with emphasis on device setup and results from validation procedures.	Parkinson disease patients (aged > = 18 years of age)	Average per study = 44 participants Total = 177 participants	*N* = 2 (accelerometer) *N* = 1 accelerometer and gyroscope) *N* = 1 (accelerometer and force sensor)	High specificity (86.4–98.6%) and sensitivity (93.1% only one study) for wearable device detection of falls.
**Rucco et al 2018** [11]	*N* = 42 (2002–2017)	IEEE Xplore SpringerLink Science Direct PubMed	To provide an overview of the most adopted sensing technologies in these fields, with a focus on the type of sensors (rather than algorithms), their position on the body and the kind of tasks they are used in.	Healthy “aged” population	Average per study = 32 participants Total = 1331 participants	*N* = 12 (accelerometer) *N* = 7 (accelerometer and gyroscope) *N* = 6 (accelerometer and pressure sensors) *N* = 3 (accelerometer + another device) *N* = 1 (gyroscope) *N* = 4 (camera or radar or console) *N* = 9 (three or more devices)	• Single sensor = 70% use accelerometer • Two sensors = 1) Approaches that combine accelerometer with a pressure sensor (usually in shoes). 2) Approaches that use accelerometer and gyroscope sensors (usually on same electronic board). • Three or more sensors = other sensing technology used (magnetometer, camera, EMG). • Sensor placement = mainly on the trunk. Second most likely position is foot or leg (about 30%).
**Sun et al 2018** [14]	*N* = 22 (2011–2017)	PubMed Web of Science Cochrane Library CINAHL	To systematically evaluate the use of technology in performing fall risk assessments, and more specifically, to evaluate the test, sensor, and algorithm effectiveness on predicting and/or discriminating older adult fallers from non-fallers.	Older adults (Aged > 60 years of age)	Average per study = 86 participants Total = 1896 participants	*N* = 11 (accelerometer) *N* = 4 (accelerometer and gyroscope) *N* = 4 (console) *N* = 1 (laser) *N* = 2 (accelerometer and pressure sensor)	A diverse range of diagnostic performance was observed (Accuracy: 47.9–100%, Sensitivity: 16.7–100%, Specificity: 40–100%, AUC 0.65–0.89) for wearable device detection of falls.

### Data synthesis

Aggregated data was used to undertake a narrative synthesis, and this was used to describe and evaluate the body of literature and tabulated in an excel spreadsheet (see Table 1). Additional meta-analysis was not possible due to large heterogeneity between included studies. The narrative synthesis was based on the extracted data and was drafted by the 1st reviewer with the 2nd reviewer checking the data synthesis of the 1st reviewer. Any discrepancies were resolved by consensus discussion chaired by the 3rd reviewer with the 3rd reviewer making the final decision.

### Risk of Bias and relative quality assessment

Two reviewers independently assessed the methodological quality of the included systematic reviews using the AMSTAR2 checklist for systematic reviews [20]. Any discrepancies were resolved by consensus discussion chaired by the 3rd reviewer with the 3rd reviewer making the final decision.

This paper includes a summary of the findings of the relative quality assessment (see Table 2) for transparency and to reveal the methodological issues in the included systematic reviews that future studies in this field should take into consideration when producing their articles in order to produce more valid scientific evidence.

**Table 2:** Results of the Relative Quality Assessment of the Included Systematic Reviews

Study	Q1	Q2	Q3	Q4	Q5	Q6	Q7	Q8	Q9	Q10	Q11	Q12	Q13	Q14	Q15	Q16	Overall Quality of Study
Pang et al. 2019 [17]	Y	Y	N	Y	N	Y	N	Y	PY	N	N/A	N/A	Y	Y	N/A	Y	**Moderate**
Nguyen et al. 2018 [13]	Y	N	N	Y	N	Y	N	PY	N	N	N/A	N/A	N	N	N/A	Y	**Critically low**
Montesinos et al. 2018 [10]	Y	N	Y	Y	N	Y	Y	Y	Y	N	Y	Y	Y	Y	Y	Y	**Moderate**
Chaudhuri et al. 2014 [16]	Y	N	N	Y	Y	N	N	N	Y	N	N/A	N/A	N	N	N/A	N	**Low**
Silva de Lima et al. 2017 [15]	Y	N	N	Y	N	N	N	N	N	N	N/A	N/A	N	N	N/A	Y	**Critically low**
Rucco et al. 2018 [11]	Y	N	N	Y	N	N	N	Y	N	N	N/A	N/A	N	Y	N/A	Y	**Critically low**
Sun et al. 2018 [14]	Y	N	Y	Y	N	N	N	PY	N	N	N/A	N/A	N	Y	N/A	Y	**Critically low**

This relative quality assessment tool follows the AMSTAR2 checklist [20]. This scale has four ratings for systematic reviews: critically low, low, moderate, high

*Y* Yes, *PY* Partial Yes, *N* No, *NA* Not applicable

AMSTAR 2 [20] is a commonly used instrument for critically appraising systematic reviews and looks at 16 items in total. AMSTAR 2 does not generate an overall score but generates a rating of overall confidence: critically low, low, moderate, and high. The relative quality assessment of studies will be considered in my discussion and conclusion.

## Results

### Studies included

Following the search strategy described above, 12 records were identified through database searching and 18 records were identified through other sources (grey literature search, checking reference lists, as well as forward citation searching). After removing four duplicates, 26 records were screened by title and abstract and 15 records were removed after screening the titles and abstracts against the inclusion/exclusion criteria, which left 11 papers to be read in full text.

**Fig. 1:**
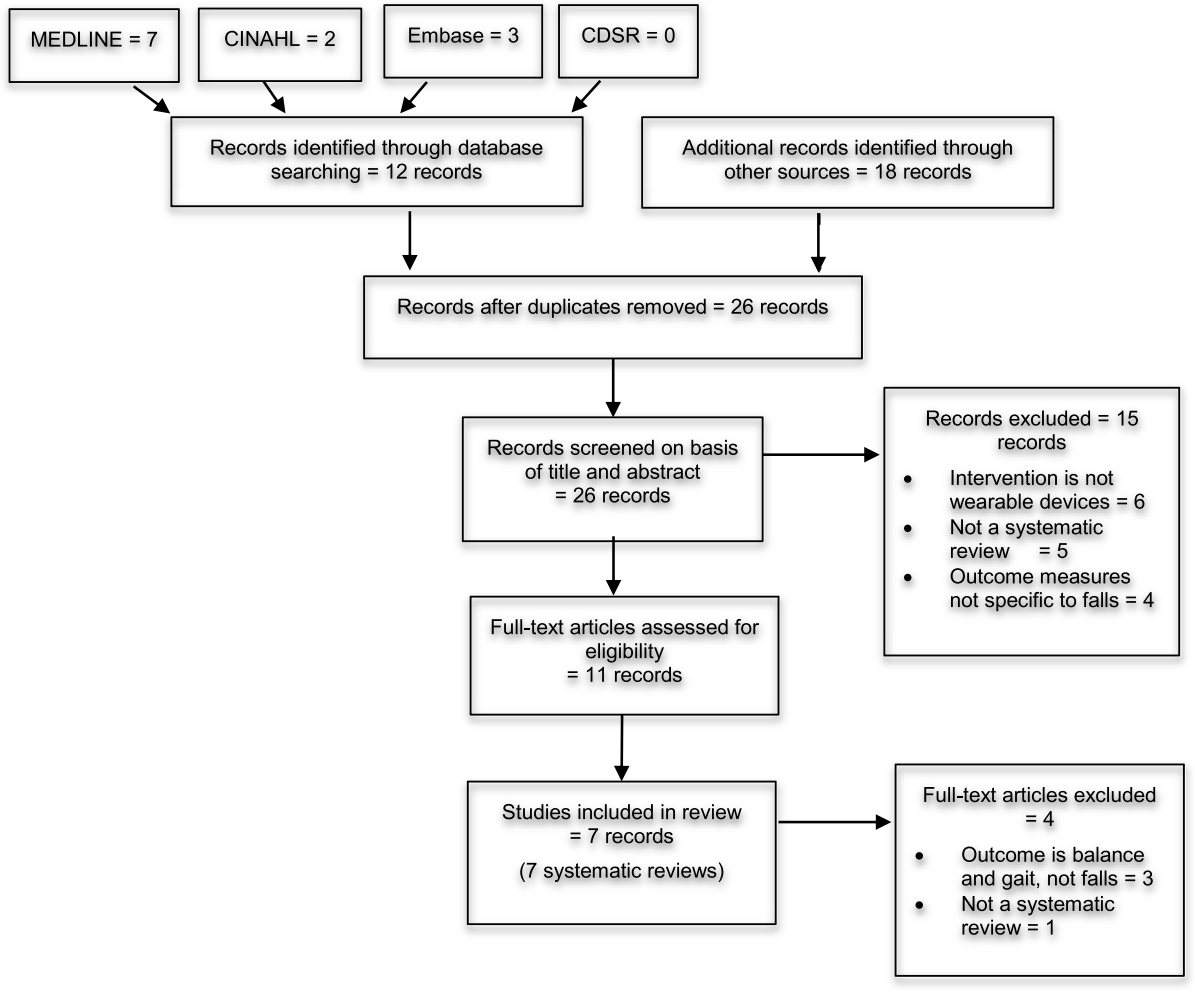
PRISMA flowchart outlining the study selection process (Adapted from the PRISMA statement [18])

After reading the full-text, four papers were excluded due to inclusion/exclusion criteria. Wang et al [21] was excluded from full-text review due to not being a systematic review. Importantly, three reviews [22–24] were excluded on full-text review as their outcome measures were not relevant. Therefore, seven papers were included in this review [13–17, 25, 26]. A flowchart of the study selection process is shown in Fig. 1.

### Characteristics of included systematic reviews

The seven included systematic reviews included from four to 57 studies (mean ± standard deviation: 24.43 ± 17.57 participants), which were relevant to the review questions, giving a cumulative number of studies of 161 (see Table 1). Chaudhuri et al [16] included 82 articles; however, only 57 articles met the inclusion criteria of using wearable devices as the intervention and so 35 articles from this systematic review were not included in this umbrella review. Silva de Lima [15] included 27 articles with 4 studies meeting the inclusion criteria of this umbrella review.

All the studies included adults (aged > = 18 years of age) only and did not include any studies that investigated fall detection in children. There was a varied success in the reporting of demographic details about individuals in the included studies and so it was not possible to extract meaningful data about the demographics of individuals included in the studies within the included systematic reviews. Montesinos et al [10] had more strict population criteria with the exclusion of patients with severe cognitive or motor impairment. Silva de Lima [15] only included patients with a diagnosis of Parkinson’s disease.

The average number of participants per study included in the systematic reviews ranged from seven to 93 participants per study (mean ± standard deviation: 49.17 ± 35.01 participants per study). The total number of participants in all included studies within each systematic review ranged from 170 to 1896 total participants (mean ± standard deviation: 829.50 ± 683.32 total number of participants). Chaudhuri et al [16] provided no information about the sample sizes in their included studies.

Five reviews only included articles in which the full-text was available in English [10, 13–17]. Montesinos et al. included articles written in English, Italian, Spanish or French [10]. Rucco et al. did not report their language restrictions for inclusion/exclusion; however, the 42 included articles were all available as full-text articles in English [11].

### Types of wearable devices in included systematic reviews

Accelerometers were the most commonly used type of device used in the included reviews for falls detection. Out of the 161 included studies, 43 studies used accelerometers only and another 34 studies used accelerometers in combination with other technology. The most commonly used combination was accelerometer and gyroscope devices (20 studies). Other types of devices were also used, and these include camera/laser (11 studies), accelerometer and pressure/force sensors (9 studies), consoles (4–8 studies), wireless networks (2 studies) and three or more devices in combination (13 studies). Chaudhuri et al. [16] did not provide specific data on types of wearable devices.

### Wearable devices for falls detection and their effectiveness

Due to heterogeneity in the methods between the included systematic reviews and their reporting of results measuring different outcomes, a meta-analysis could not be performed.

Three systematic reviews reported an average sensitivity of 93.1% or greater and an average specificity of 86.4% or greater [15–17]. Another systematic review reported a large range for sensitivity between 16.7–100% and a large range for specificity between 40 and 100% [14]. Three studies did not report sensitivity or specificity data [11, 13, 16]. Accuracy data were too heterogeneous and under-reported to comment on. Four studies compared wearable device locations and found that the trunk, lower back and foot or leg were the most accurate [10, 11, 16, 17]. One systematic review found that accuracy was improved by increasing the number of wearable devices [17].

### Quality appraisal methods of studies included within included systematic reviews

Five of the included systematic reviews made no attempt to assess the risk of bias in the individual studies they included (See Table 2) [11, 13–16]. Pang et al [17] used a self-designed relative quality assessment tool for included studies which, although cannot be validated, seemed comprehensive. Pang et al. ranked their studies out of 7 which resulted in a median score = 3/7 and an average score of 2.6/7 (low to moderate quality studies). Montesinos et al [10] used a checklist adapted from Downs and Black for included studies. This checklist found that there was external validity for all included studies; however, the internal validity of 6 (out of 13) of the included studies was unclear due to unreported variables.

### Quality appraisal of included systematic reviews

The seven systematic reviews included in this umbrella review were assessed by the AMSTAR2 [20] checklist which ranks systematic reviews from critically low, low, moderate and high quality. Four systematic reviews [11, 13–15] were ranked critically low quality, meaning that there is more than one critical flaw and should not be relied on to provide an accurate and comprehensive summary of the available studies. One systematic review, Chaudhuri et al [16], was ranked low quality which means that it has one critical flaw and may not provide an accurate and comprehensive summary of the available studies that address the question of interest. Two systematic reviews [10, 17] were ranked moderate quality which means that there is more than one weakness, but no crucial flaws, and it may provide an accurate summary of the results of the available studies that were included in the review.

All the systematic reviews asked appropriate research questions (covered PICO) and had a comprehensive literature search strategy. Only Pang et al [17], reported a protocol established before the conduct of their review. Only Chaudhuri et al [16] reported performing their study selection in duplicate; however, some systematic reviews may have done this but not reported it in their final paper.

Montesinos et al [10] was the only systematic review that performed a meta-analysis. The other systematic reviews cited heterogeneity in study designs and outcome measures as the reasons for being unable to undertake a meta-analysis. Other common limitations of studies were low sample sizes and a lack of peer-review.

## Discussion

This umbrella review summarised the scientific literature focussing on the use of wearable electronic devices for falls detection and prevention. Three of the included reviews focussed mainly on falls detection, two focussed mainly on falls risk assessment, one focussed on falls management and one focussed on assessing the most widely adopted technologies in this field [10, 11, 13–16, 27].

### Summary of evidence

Most reviews reported that wearable devices are an effective, low-cost tool for detecting falls and sending a signal to call for help. The most effective sensors are placed on the trunk or foot/leg with multiple sensors increasing the accuracy, specificity, and sensitivity of these devices. However, these results must be viewed cautiously as many reviews reported a lack of high-quality studies in the field and a lack of “real world” testing of these devices in older people. The included reviews also call for “nonobstructive” devices that are low-cost and maintain users’ privacy. The use of wearable devices as part of a falls risk assessment has, yet not been validated but is another potential future use of these devices.

The evidence with regards to older adults, specifically, is less clear as more studies are needed to look at detecting falls in frail older people who can be more difficult to recruit into studies. Also, the current algorithms that these devices run are quite accurate, but more work is needed here as it is vital to reduce false-positive rates with these devices to avoid ‘alarm fatigue’.

Montesinos et al [10] was rated highly as a moderate quality systematic review and was the only systematic review to perform a meta-analysis. The statistical analysis reported significant, very strong, positive associations in three different triads of feature category, task, and sensor placement:

- Angular velocity – walking – shins.
- Linear acceleration – quiet standing – lower back
- Linear acceleration – stand to sit/sit to stand – lower back

Montesinos el at recommended these as the optimal combinations when using wearable devices to discriminate between fallers and non-fallers. Furthermore, they found four statistically significant features that were observed with fallers which included: step time, Coefficient of Variation (CV) for step time, CV for stride time, CV for clinical support time. These statistically significant findings should be considered when developing a standardized, valid evaluation tool for these devices that this umbrella review recommends to future researchers. It is important to note that there are lots of studies on wearable devices for falls detection; however, there is little agreement about the best type and design of the device with regards to the type of sensor, number of sensors and a signal processing algorithm.

### Strengths of this review

This review has several strengths and is the first umbrella review of its kind. The research methods were extensive and are detailed in the method section of this review as well as a link to the protocol which was established prior to the conduct of the review. An extensive, peer-reviewed, search strategy was conducted, thoroughly searching the four most relevant bibliographic databases with no date-of-publication restrictions. This paper includes a comprehensive quality assessment of the included systematic reviews. Therefore, this umbrella review provides a comprehensive and methodologically strong overview of the currently published research on this topic. The PRISMA checklist can be found attached as Appendix 2.

### Limitations of this review

This umbrella review must be interpreted within the context of its limitations. Firstly, this review is at risk of language bias since this review is based exclusively on studies reported in English. However, all studies found through searching were available in English. Furthermore, there is potential that publication bias has hidden potentially relevant trials and their results from this review. The effect of this should be limited by the extensive search strategy and the fact that none of the authors has declared any competing interests with this review.

The main limitations of this review come from major methodological weaknesses in the included systematic reviews. Only two reviews [10, 17] were ranked as moderate quality with the other reviews ranking as low or critically low quality. Common problems in the methods of the included systematic reviews include no protocol established prior to the conduct of the review, not performing study selection and data extraction in duplicate and no risk of bias assessment for individual studies that were included in the included reviews.

Only Montesinos et al [10] was able to conduct a meta-analysis and the other systematic reviews were not able to due to the often small number of included studies and heterogeneous methodologies of those studies. Heterogeneity in included studies mainly stems from the fact there is not a validated way to evaluate wearable devices, with lots of different outcome measures that make it difficult to draw conclusions from. There was also much variation in the studies in measured parameters, assessment tools, sensor sites, tasks and assessing falls.

### Implications for future research

The literature in this field is still in its infancy and more high-quality studies are needed. Rucco et al. demonstrated that the topics of risk assessment, falls monitoring, and falls prevention in older people are of increasing interest to researchers, with “an almost linear growth of the published manuscripts” [11].

The heterogeneity in the study designs has been discussed at length in this review and must be standardized for future reviews. There needs to be a set of validated outcomes when assessing these devices for falls detection that are agreed upon and used as the standard in future research.

A recent Cochrane review described that most studies in this field fail to specify a definition of falls; thus, leaving the interpretation to study participants and researchers [28]. Due to the heterogeneity in the interpretation of “a fall”, the validity of the studies could be brought into question. This umbrella review found many different interpretations of falls in the included systematic reviews and, in addition to the evidence in the Cochrane review, would strongly recommend that future studies provide an operational definition of a fall with clear inclusion/exclusion criteria.

This umbrella review has revealed some important questions and areas of interest that researchers in this field should investigate:

- Are wearable devices as effective as proven in previous studies if tested in “real world” settings with a large sample size of older adults?
- What is the most effective system design that older adults will accept for use in daily living?
- Can wearable devices be used to enable alerts of deteriorating balance control?
- How, practically, could wearable devices be integrated with a comprehensive falls risk assessment?
- How can the gap between clinical functionality and user experience of these devices be improved?
- An effective, validated, tool for evaluating wearable devices for falls detection that can be replicated in future high-quality studies.
- What are the most effective algorithms to use combined with these wearable technologies?
- Is there potential for these devices to be used in different types of falls experienced by people with stroke, MS, age-related frailty, and other conditions associated with ageing?

### Implications for practice

In order to recommend widespread implementation, healthcare providers need more evidence that assesses the cost-benefit that these devices provide and how they could be implemented on a large-scale.

These devices are accurate and low-cost and may be increasingly purchased by individuals. Personal emergency response systems (PERS) are a currently commonly used commercial solution to issues like this and allow a way for individuals to press a button and contact an emergency centre [29]. Wearable devices for falls detection have an advantage in that they will still call for help if the user is rendered unable to do so themselves as it does not rely on the user pressing a button. This is particularly important given that a recent cohort study found that up to 4/5 older adults wearing PERS did not activate it to call for assistance when they had a fall [30].

## Conclusions

This review has demonstrated that wearable device technology is effective at detecting falls and is a promising emerging field of telemedicine that can offer a low-cost and accurate way to detect falls and summon for help. Their use for falls prevention needs to be further examined with the literature showing promise for their use as part of a falls risk assessment which then can be used to categorise risk and guide interventions. This review also found that there are significant differences in the effectiveness of these devices depending on the type of device and where it is placed on the body. The current evidence would suggest that researchers should be testing these devices on the trunk of the body or on the legs/shin and most devices use accelerometers, often in combination with gyroscopes.

Further high-quality studies in this field are needed and researchers should consider common flaws reported in this review. This review found significant heterogeneity in the study designs and methods between reviews and studies. Several studies reported difficulties in recruiting older adults; however, testing these devices on older adults in ‘real-world environments’ is essential if we are going to understand their effectiveness for older adults. A standardized evaluation tool for wearable devices with standardised outcome measures would improve the validity of research in this field. Older adults have been reported to want a low-cost device which they can understand how it works and which is highly accurate.

## Data Availability

Not applicable.

## Appendix 1

### MEDLINE (main search strategy)

1. Wearable Electronic Devices/
2. wearable electronic device.kw.
3. wearable device.kw.
4. (wear$4 adj3 electronic adj device$1). ab, ti.
5. (wear$4 adj3 technolog$3). ab, ti.
6. (wear$4 adj3 device$1). ab, ti.
7. (wear$4 adj3 sensor$1). ab, ti.
8. (smart adj watch$2). ab, ti.
9. 1 or 2 or 3 or 4 or 5 or 6 or 7 or 8
10. Accidental Falls/pc [Prevention & Control]
11. Accidental Falls/
12. Accident Prevention/
13. 11 and 12
14. accidental falls.kw.
15. falls prevention.kw.
16. (fall$3 adj2 incidence$1). ab, ti.
17. (fall$3 adj2 prevent$3). ab, ti.
18. (accidental$2 adj1 fall$3). ab, ti.
19. 10 or 13 or 14 or 15 or 16 or 17 or 18
20. 9 and 19
21. Meta-Analysis as Topic/
22. meta analy$.tw.
23. metaanaly$.tw.
24. Meta-Analysis/
25. (systematic adj (review$1 or overview$1)).tw.
26. exp. Review Literature as Topic/
27. 21 or 22 or 23 or 24 or 25 or 26
28. cochrane.ab.
29. cochrane.ab.
30. (psychlit or psyclit). ab.
31. (psychinfo or psycinfo). ab.
32. (cinahl or cinhal). ab.
33. science citation index.ab.
34. bids.ab.
35. cancerlit.ab.
36. 28 or 29 or 30 or 31 or 32 or 33 or 34 or 35
37. reference list$. ab.
38. bibliograph$. ab.
39. bibliograph$. ab.
40. relevant journals.ab.
41. manual search$. ab.
42. 37 or 38 or 39 or 40 or 41
43. selection criteria.ab.
44. data extraction.ab.
45. 43 or 44
46. Review/
47. 45 and 46
48. Comment/
49. Letter/
50. Editorial/
51. animal/
52. human/
53. 51 not (51 and 52)
54. 48 or 49 or 50 or 53
55. 27 or 36 or 42 or 47
56. 55 not 54
57. 20 and 56

## Appendix 2

**Table 3:** PRISMA Checklist

Section/topic	#	Checklist item	Reported on page #
**TITLE**			
Title	1	Identify the report as a systematic review, meta-analysis, or both.	0
**ABSTRACT**			
Structured summary	2	Provide a structured summary including, as applicable: background; objectives; data sources; study eligibility criteria, participants, and interventions; study appraisal and synthesis methods; results; limitations; conclusions and implications of key findings; systematic review registration number.	0
**INTRODUCTION**			
Rationale	3	Describe the rationale for the review in the context of what is already known.	3–5
Objectives	4	Provide an explicit statement of questions being addressed with reference to participants, interventions, comparisons, outcomes, and study design (PICOS).	6–7
**METHODS**			
Protocol and registration	5	Indicate if a review protocol exists, if and where it can be accessed (e.g., Web address), and, if available, provide registration information including registration number.	5
Eligibility criteria	6	Specify study characteristics (e.g., PICOS, length of follow-up) and report characteristics (e.g., years considered, language, publication status) used as criteria for eligibility, giving rationale.	9–10
Information sources	7	Describe all information sources (e.g., databases with dates of coverage, contact with study authors to identify additional studies) in the search and date last searched.	5–6
Search	8	Present full electronic search strategy for at least one database, including any limits used, such that it could be repeated.	24–25
Study selection	9	State the process for selecting studies (i.e., screening, eligibility, included in systematic review, and, if applicable, included in the meta-analysis).	7
Data collection process	10	Describe method of data extraction from reports (e.g., piloted forms, independently, in duplicate) and any processes for obtaining and confirming data from investigators.	7–8
Data items	11	List and define all variables for which data were sought (e.g., PICOS, funding sources) and any assumptions and simplifications made.	9–11
Risk of bias in individual studies	12	Describe methods used for assessing risk of bias of individual studies (including specification of whether this was done at the study or outcome level), and how this information is to be used in any data synthesis.	11
Summary measures	13	State the principal summary measures (e.g., risk ratio, difference in means).	9–11
Synthesis of results	14	Describe the methods of handling data and combining results of studies, if done, including measures of consistency (e.g., I^2^) for each meta-analysis.	9–11
Risk of bias across studies	15	Specify any assessment of risk of bias that may affect the cumulative evidence (e.g., publication bias, selective reporting within studies).	15
Additional analyses	16	Describe methods of additional analyses (e.g., sensitivity or subgroup analyses, meta-regression), if done, indicating which were pre-specified.	8–12
**RESULTS**			
Study selection	17	Give numbers of studies screened, assessed for eligibility, and included in the review, with reasons for exclusions at each stage, ideally with a flow diagram.	8–11
Study characteristics	18	For each study, present characteristics for which data were extracted (e.g., study size, PICOS, follow-up period) and provide the citations.	8–12
Risk of bias within studies	19	Present data on risk of bias of each study and, if available, any outcome level assessment (see item 12).	34–35
Results of individual studies	20	For all outcomes considered (benefits or harms), present, for each study: (a) simple summary data for each intervention group (b) effect estimates and confidence intervals, ideally with a forest plot.	8–12
Synthesis of results	21	Present results of each meta-analysis done, including confidence intervals and measures of consistency.	N/A
Risk of bias across studies	22	Present results of any assessment of risk of bias across studies (see Item 15).	11–12
Additional analysis	23	Give results of additional analyses, if done (e.g., sensitivity or subgroup analyses, meta-regression [see Item 16]).	N/A
**DISCUSSION**			
Summary of evidence	24	Summarize the main findings including the strength of evidence for each main outcome; consider their relevance to key groups (e.g., healthcare providers, users, and policy makers).	13–17
Limitations	25	Discuss limitations at study and outcome level (e.g., risk of bias), and at review-level (e.g., incomplete retrieval of identified research, reporting bias).	15
Conclusions	26	Provide a general interpretation of the results in the context of other evidence, and implications for future research.	16–17
**FUNDING**			
Funding	27	Describe sources of funding for the systematic review and other support (e.g., supply of data), role of funders for the systematic review.	18–19

*PRISMA Checklist 2009:* Moher D, Liberati A, Tetzlaff J, Altman DG, The PRISMA Group (2009). Preferred Reporting Items for Systematic Reviews and Meta-Analyses: The PRISMA Statement. PLoS Med 6(6): e1000097. doi:10.1371/journal.pmed1000097
